# Arrhythmogenic Remodeling of the Left Ventricle in a Porcine Model of Repaired Tetralogy of Fallot

**DOI:** 10.1161/CIRCEP.117.006059

**Published:** 2018-10-10

**Authors:** Virginie Dubes, David Benoist, François Roubertie, Stephen H. Gilbert, Marion Constantin, Sabine Charron, Delphine Elbes, Delphine Vieillot, Bruno Quesson, Hubert Cochet, Michel Haïssaguerre, Caroline Rooryck, Pierre Bordachar, Jean-Benoit Thambo, Olivier Bernus

**Affiliations:** 1 IHU LIRYC, L’Institut de Rythmologie et Modélisation Cardiaque, Fondation Bordeaux Université, Pessac, France (V.D., D.B., F.R., S.H.G., M.C., S.C., D.E., B.Q., H.C., M.H., C.R., P.B., J.-B.T., O.B.).; 2 Inserm U1045, Centre de Recherche Cardio-Thoracique de Bordeaux, Université de Bordeaux, France. (V.D., D.B., F.R., S.H.G., M.C., S.C., D.E., B.Q., H.C., M.H., P.B., J.-B.T., O.B.),; 3 Plateforme Technologique d’Innovation Biomédicale, Université de Bordeaux, France. (D.V.); 4 Inserm U1211, Maladies Rares: Génétique et Métabolisme, Université de Bordeaux, France. (C.R.); 5 Centre Hospitalier Universitaire de Bordeaux, Hôpital Cardiologique du Haut-Lévêque, Pessac, France (F.R., H.C., M.H., C.R., P.B., J.-B.T.).; 6 Max Delbröck Center for Molecular Medicine, Berlin, Germany (S.H.G.).; 7 Institute of Biomedical Engineering, University of Oxford, United Kingdom (D.E.).

**Keywords:** arrhythmias, disease model, heart ventricles, swine, tetralogy of Fallot

## Abstract

**Background:**

Ventricular arrhythmias are frequent in patients with repaired tetralogy of Fallot (rTOF), but their origin and underlying mechanisms remain unclear. In this study, the involvement of left ventricular (LV) electrical and structural remodeling was assessed in an animal model mimicking rTOF sequelae.

**Methods:**

Piglets underwent a tetralogy of Fallot repair–like surgery (n=6) or were sham operated (Sham, n=5). Twenty-three weeks post-surgery, cardiac function was assessed in vivo by magnetic resonance imaging. Electrophysiological properties were characterized by optical mapping. LV fibrosis and connexin-43 localization were assessed on histological sections and protein expression assessed by Western Blot.

**Results:**

Right ventricular dysfunction was evident, whereas LV function remained unaltered in rTOF pigs. Optical mapping showed longer action potential duration on the rTOF LV epicardium and endocardium. Epicardial conduction velocity was significantly reduced in the longitudinal direction in rTOF LVs but not in the transverse direction compared with Sham. An elevated collagen content was found in LV basal and apical sections from rTOF pigs. Moreover, a trend for connexin-43 lateralization with no change in protein expression was found in the LV of rTOFs. Finally, rTOF LVs had a lower threshold for arrhythmia induction using incremental pacing protocols.

**Conclusions:**

We found an arrhythmogenic substrate with prolonged heterogeneous action potential duration and reduced conduction velocity in the LV of rTOF pigs. This remodeling precedes LV dysfunction and is likely to contribute to ventricular arrhythmias and sudden cardiac death in patients with rTOF.

## Abstract 2

Supplemental Digital Content is available in the text.

**WHAT IS KNOWN?**

- Patients with repaired tetralogy of Fallot (rTOF) experience life-threatening arrhythmias late after surgery. Surgical scars have been shown to support macroreentrant circuits and monomorphic ventricular tachycardia.
- We recently evidenced the presence of a global remodeling of right ventricular structure and electrophysiology, which contributes to arrhythmias in an rTOF porcine model.

**WHAT THE STUDY ADDS?**

- A marked remodeling of left ventricular structure and electrophysiology was found in an rTOF preclinical model with preserved left ventricular ejection fraction.
- Repolarization remodeling in the rTOF left ventricular involved an increase in action potential duration and dispersion, and slow conduction was associated with connexin-43 lateralization and fibrosis.
- This remodeling generates a proarrhythmic substrate likely to contribute to arrhythmias in rTOF.

Tetralogy of Fallot (TOF) is the most common cyanotic congenital heart disease occurring in ≈1 in 3000 births.^1^ It is characterized by subpulmonary stenosis, a subaortic ventricular septal defect, dextroposition of aorta, and right ventricular (RV) hypertrophy.^2^ Intracardiac repair has excellent short-term outcomes, but the incidence of late complications increases in parallel with a growing adult survivor population,^3^ including ventricular arrhythmia and sudden cardiac death.^4^

Prior studies have reported on the impact of functional and electrophysiological modifications focusing mainly on the RV in patients with repaired TOF (rTOF).^5^ Pulmonary insufficiency is the most common postoperative complication in patients with rTOF leading to the chronic RV volume overload and RV dysfunction.^6^ RV dilatation has been associated with QRS prolongation^7^ and the risk of malignant ventricular arrhythmias and sudden death.^5,8^ Moreover, areas of dense fibrosis owing to surgical incisions are known to determine reentry circuits, which are thought to be the main arrhythmic mechanism in these patients.^9,10^ In a porcine model of rTOF, we have shown a remodeling of RV conduction and repolarization, which extended well beyond the scar region and was likely to contribute to rTOF arrhythmogenicity.^11,12^

There is recent evidence showing that patients with rTOF can also present altered left ventricular (LV) function and tissue structure as a consequence of ventricular-ventricular interaction.^13–16^ Furthermore, isolated RV pressure overload because of pulmonary hypertension was found to promote an electrophysiological remodeling of the LV.^17,18^ Despite these observations, LV electrophysiological remodeling and its implication in rTOF arrhythmogenicity remains unknown.

In this study, we demonstrated the presence of a proarrhythmic electrical and structural remodeling in the LV of a porcine model of rTOF in the absence of significant alterations in LV function. We found that fibrosis is associated with significant electrophysiological alterations, including conduction slowing and increased dispersion of repolarization, which could play a major role in the onset and maintenance of ventricular arrhythmias in rTOF.

## Methods

The data, analytic methods, and study materials will not be made available to other researchers for purposes of reproducing the results or replicating the procedure.

An expanded Methods section is available in the Data Supplement.

### Animal Model

Large, white newborn piglets (<12 kg) were used in accordance with the European Union Council Directive 2010/63/EU for the protection of animals used for scientific purposes and with local ethical committee approval. Animals (rTOF, n=6) were sedated with ketamine (10 mg/kg, intramuscular, Vibrac) and acepromazine (0.1 mg/kg, intramuscular, Vetoquinol). Anesthesia was induced with sodium pentobarbital (5 mg/kg, intravenous Ceva) and maintained with isoflurane (2% in 100% oxygen, Vibrac). After a left lateral thoracotomy, the pericardium was opened, the pulmonary artery was clamped longitudinally using a side-biting clamp to avoid RV outflow tract obstruction, and a 2-cm incision was made across the pulmonary annulus. Two pulmonary valve leaflets were then excised before closing the back the incision with a polytetrafluorethylene patch. A Goretex tape was loosely tied (2 cm diameter) around the pulmonary artery 1 cm distal to the valve annulus. Control animals (Sham, n=5) underwent a sham operation consisting of a left lateral thoracotomy followed by an opening of the pericardium with no further intervention. Animals were then studied at 23±1 weeks post-surgery.

### Cardiac Magnetic Resonance

Animals were sedated and anaesthetized as explained above. A cardiac magnetic resonance exam was performed using a Siemens Magnetom Avanto 1.5T MRI scanner (Erlangen, Germany).

After magnetic resonance imaging acquisition, pigs were euthanized by injection of sodium pentobarbital (10 mL from 200 mg/mL stock), and their hearts were rapidly excised.

### Optical Mapping of Anterior LV Wedges

The anterior LV was dissected, and the left anterior descending coronary artery was cannulated. The wedge was installed in a bath (37°C) and perfused at 20 mL/min with a modified Krebs-Henseleit solution. After electromechanical uncoupling using blebbistatin (10 µmol/L; Enzo Life Sciences), the preparation was loaded with the voltage-sensitive dye di-4-ANEPPS (10 µmol/L; Biotium). Fluorescence emitted from both epicardial and endocardial surfaces was recorded with 2 CMOS cameras (SciMedia USA Ltd) at 1 mm spatial resolution.

The wedges were incrementally paced from 1 to 5 Hz at the epicardial base to investigate restitution properties. Ventricular arrhythmias occurring during the dynamics restitution protocol were monitored and were classified according to their type: ventricular tachycardia or fibrillation, duration, and stimulation frequency threshold required for induction. Sustained arrhythmias were defined as lasting for at least 30 seconds and requiring external defibrillation for termination (30J). Epicardial and endocardial action potential durations (APDs) were measured at 80% of repolarization (APD_80_) in 5×5 mm regions at the base, the mid free wall, and the apex of the anterior LV. A contour was manually applied to activation and APD_80_ maps to exclude low-quality optical signals and artifacts. APD_80_ and repolarization time dispersion was quantified across the LV. The effective refractory period was measured by an S1-S2 protocol. Longitudinal conduction velocity and transverse conduction velocity were measured along the directions of the fastest and slowest propagation velocity from the stimulus point.

### Histology

Tissue samples from the LV apex and base were fixed in 4% paraformaldehyde (Sham and rTOF, n=4) and transmural sections (8 µm) stained with Masson Trichrome. Slides were examined at ×10 magnification on a Nikon Eclipse 80i. Interstitial collagen quantification was expressed as a percentage of total tissue area using Image J software.

### Immunohistochemistry

After tissue preparation steps, LV transmural sections were incubated with anti-connexin 43 antibodies (1:250; Millipore) followed by incubation with biotinylated secondary antibodies and the conjugated enzyme streptavidine peroxydase. Immunoreactive cells were stained with the 3-amino-9-ethyl-carbazole and counterstained with hematoxylin. Slides were examined at ×40 magnification on a Nikon Eclipse 80i.

### Western Blots

LV tissue samples were snap-frozen in liquid nitrogen and stored at −80°C. Proteins (50 µg) were separated on acrylamide gels (TGX Stain-Free Precast Gels; Bio-Rad) and transferred (Trans-Blot Turbo; Bio-Rad) onto polyvinylidene difluoride membranes. After incubation with primary antibodies (Connexin-43, 1:500; Sigma-Aldrich) and HRP-conjugated secondary antibodies (1:2000; Bio-Rad), total hybridized protein levels were imaged under UV light and immunospecific signals revealed by enhanced chemiluminescence (Thermo Scientific). Quantification of Western blot was done with ImageJ (National Institutes of Health).

### Data Analysis

Data are presented as mean±SD. Differences were determined using Mann-Whitney *U* tests, 1-way ANOVA with a Holm-Sidak multiple comparison test or its nonparametric equivalent, and Spearman correlation analysis as appropriate using SigmaStat software. A *P* value of <0.05 was considered significant.

## Results

### Animal Model

Heart weight normalized to body surface area (BSA) was increased in rTOF pigs 23±1 weeks post-surgery (Table). However, the LV weight-to-body weight ratio was not significantly changed, highlighting the absence of LV hypertrophy in these animal models. RV function was significantly altered in rTOF pigs as indicated by the depressed RV ejection fraction and increased end-systolic, end-diastolic volume indexed to BSA, and pulmonary regurgitation fraction (Table). According to the RV dysfunction severity grading obtained by magnetic resonance imaging in a cohort of 100 patients with rTOF,^19^ rTOF pigs had a moderate RV dysfunction (Figure I in the Data Supplement).

**Table.**
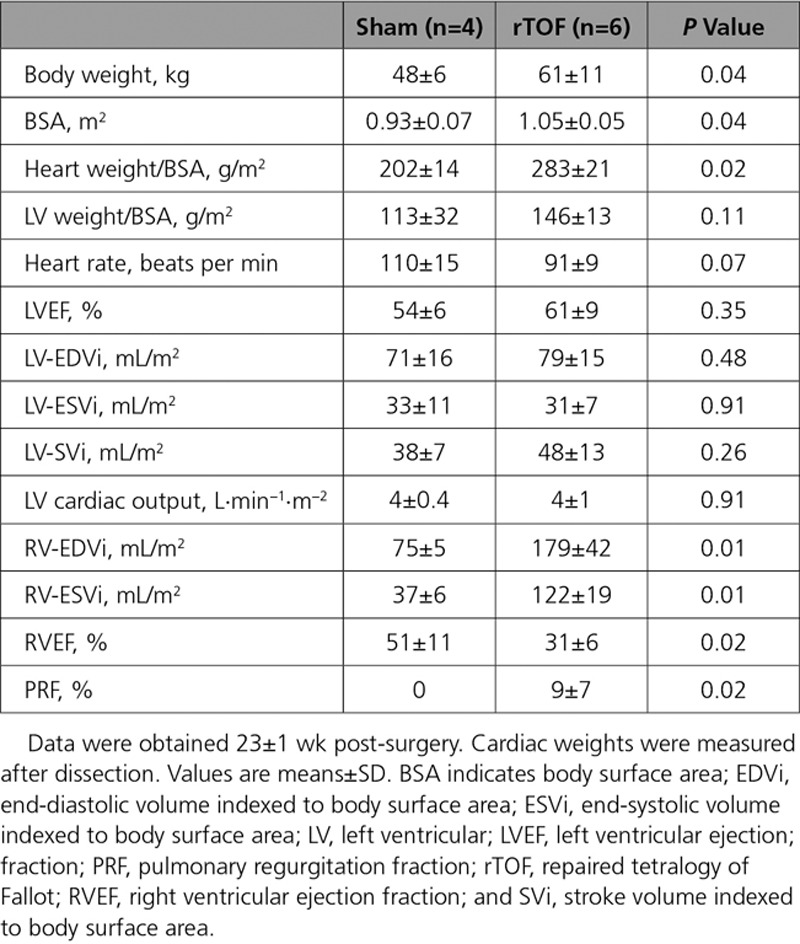
LV Function, Hemodynamics, and Anatomy

However, LV hemodynamic properties, LV ejection fraction, cardiac output, and volumes were preserved in these animals. Thus, LV function was maintained in rTOF pigs at this stage (23±1 weeks post-surgery; Table).

### LV APD, Restitution, and Dispersion

**Figure 1.**
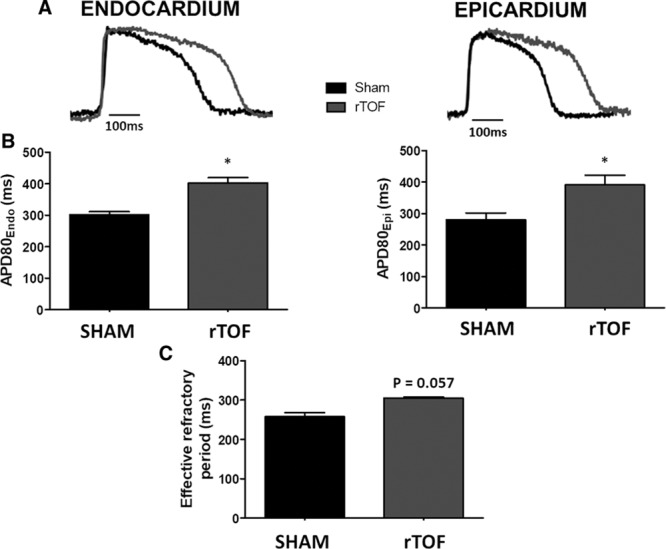
**Action potential duration (APD) from Sham and repaired tetralogy of Fallot left ventricles (LVs).** **A**, Representative optical action potentials from the LV endocardium and epicardium of Sham (black) and rTOF (grey) pigs. **B**, Endocardial and epicardial APD80 were prolonged in the anterior LV midwall of rTOF pigs (grey) compared with Sham (black) when paced at 1 Hz. **C**, LV effective refractory period tended to be longer on the rTOF epicardium than in Sham pigs. Data are means±SD. Sham, n=5; rTOF, n=6. *P<0.05.

**Figure 2.**
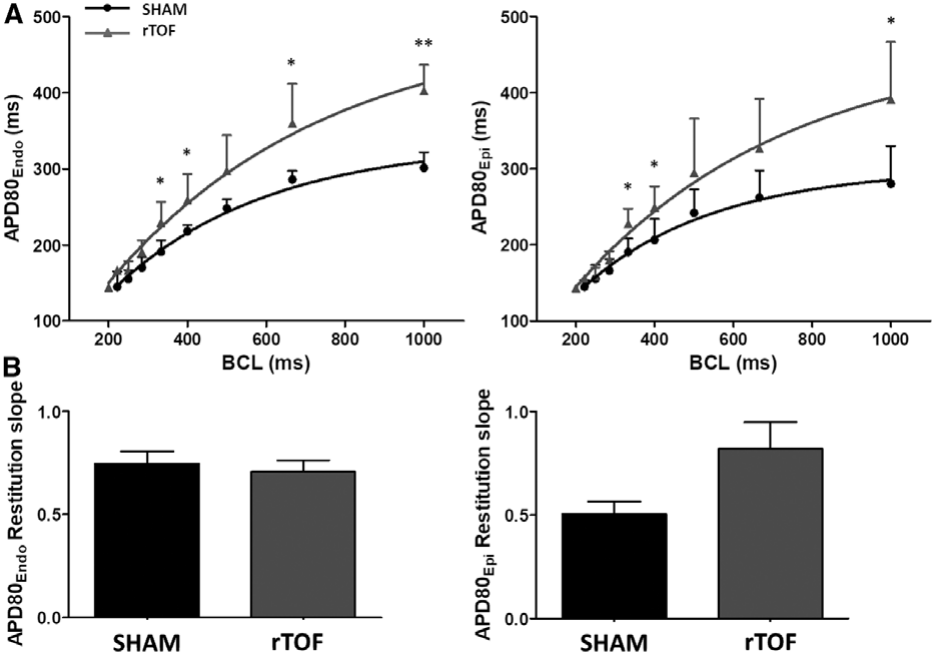
**Dynamic action potential duration (APD) restitutions in Sham and repaired tetralogy of Fallot (rTOF) left ventricles (LVs).** **A**, Mean APD80 at varying basic cycle lengths (BCL) on the LV endocardium and the epicardium. APD80 was prolonged on the epicardium and endocardium at different cycle lengths of rTOF (grey lines) and Sham (black lines) pigs. **B**, There was a trend for an increase in epicardial APD restitution slope in rTOF compared with Sham pigs. Data are means±SD. Sham, n=5; rTOF, n=6. *P<0.05, **P<0.01.

**Figure 3.**
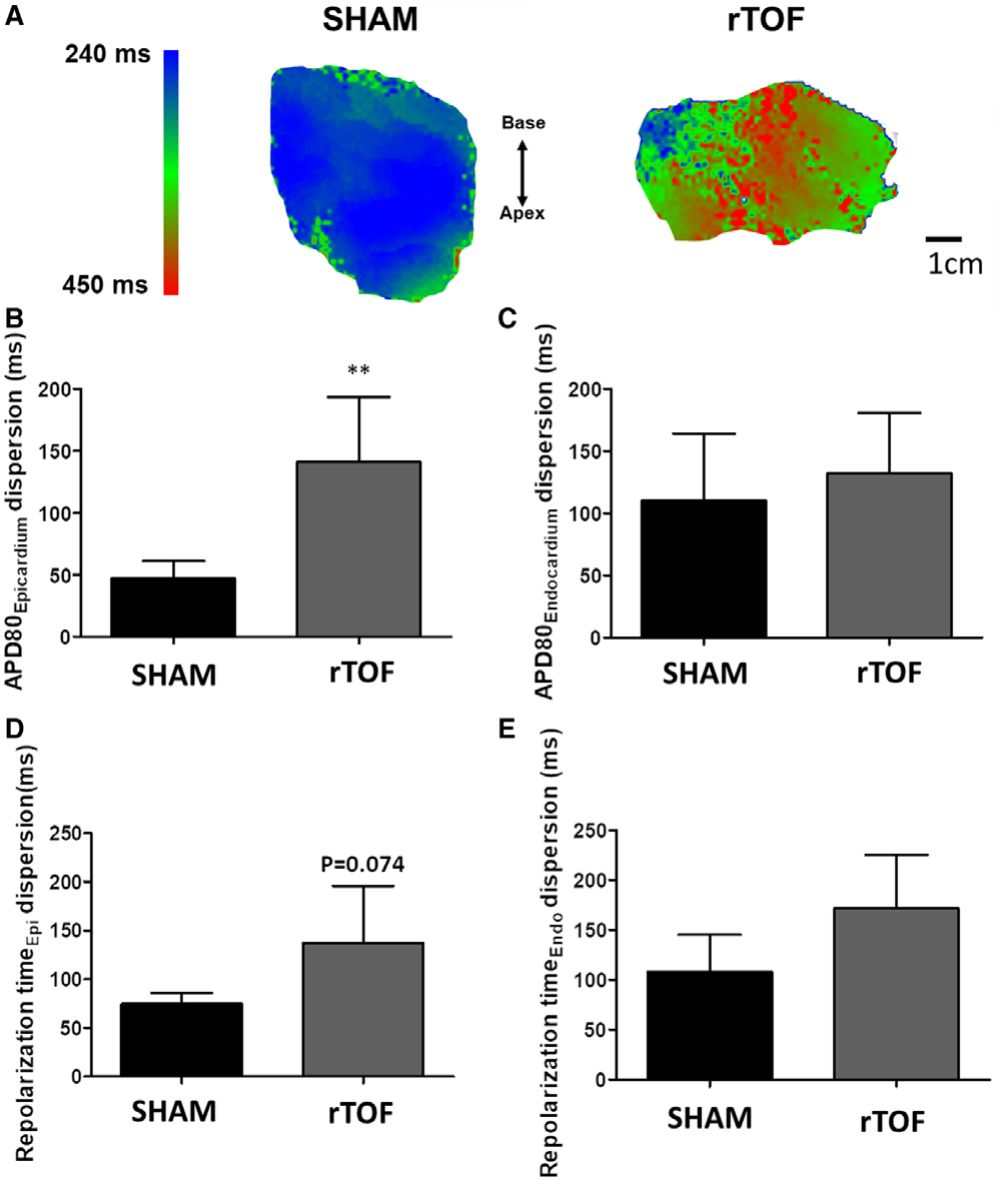
**Dispersion of repolarization in Sham and repaired tetralogy of Fallot (rTOF) left ventricles (LVs).** **A**, Representative epicardial APD80 maps showing heterogeneous action potential duration (APD) distribution in rTOF LVs paced at 1 Hz. **B**, APD80 dispersion was increased in rTOF compared with Sham in the epicardium but not the endocardium (**C**). **D**, There was a trend for an increase in repolarization time dispersion in rTOF LV epicardium but not in the endocardium (**E**). Data are means±SD. Sham, n=5; rTOF, n=6. **P<0.01.

APD_80_ measured in the midwall region of rTOF LVs was significantly prolonged both on the endocardium (403±34 versus 301±20 ms) and epicardium (390±76 versus 280±50 ms) compared with Sham (Figure 1A and 1B). In a regional analysis, we found that APD tended to be prolonged across the whole rTOF LV, but statistical significance was only reached for the midwall epicardial and endocardial regions and the apical epicardium (Figure II in the Data Supplement). There was a trend (*P*=0.057) for a prolongation of the effective refractory period obtained from the epicardium of rTOF animals (Figure 1C). APD_80_ prolongation was preserved at pacing frequencies ≤3 Hz (Figure 2A), and a trend for steeper APD restitution curve was observed on the epicardium (Figure 2B). Epicardial APD_80_ maps highlighted a heterogeneous APD distribution across the rTOF LV as opposed to the Sham (Figure 3A), and this was confirmed by APD dispersion quantification (Figure 3B). In contrast with the epicardial side, APD dispersion was not increased in rTOF LV endocardium compared with Sham pigs (Figure 3C). An increased dispersion was also found at the repolarization time level in the epicardium but not the endocardium, although this did not reach statistical significance (*P*=0.074; Figure 3D and 3E).

### LV Electrical Propagation Properties and Fibrosis

**Figure 4.**
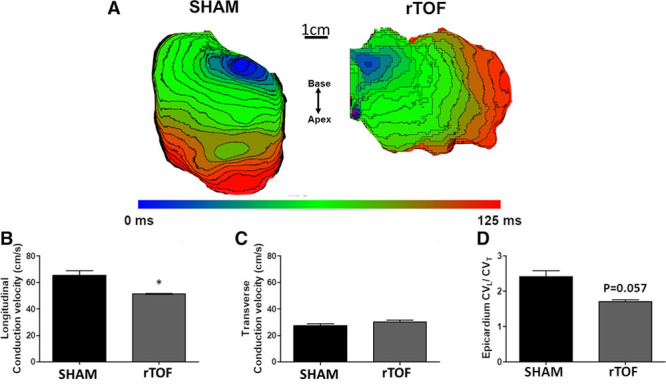
**Left ventricular (LV) activation and conduction velocity in Sham and repaired tetralogy of Fallot (rTOF) preparations.** **A**, Representative epicardial activation maps (5 ms spaced isochrones) from Sham and rTOF LVs stimulated at 1 Hz. **B**, Longitudinal conduction velocity (CV_L_) was significantly decreased in rTOF LVs compared with Sham, whereas transverse conduction velocity (CV_T_) remained unchanged (**C**). **D**, This resulted in a trend for a decreased CV_L_ to CV_T_ ratio in rTOF animals. Data are means±SD. Sham, n=4; rTOF, n=4. *P<0.05.

**Figure 5.**
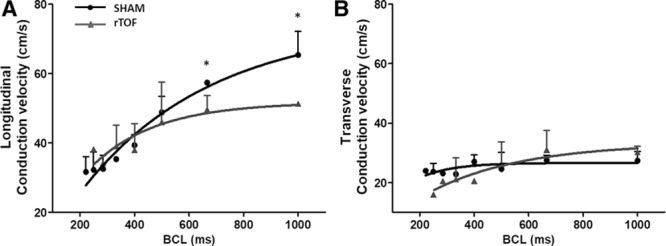
**Dynamic conduction velocity restitution curves in Sham and repaired tetralogy of Fallot (rTOF) left ventricles (LVs).** Epicardial restitution of conduction velocity measured at various cycle lengths (BCL) in the longitudinal (**A**) and transverse (**B**) directions in rTOF (grey lines) and Sham (black lines) LVs. Data are means±SD. Sham, n=4; rTOF, n=4. *P<0.05.

**Figure 6.**
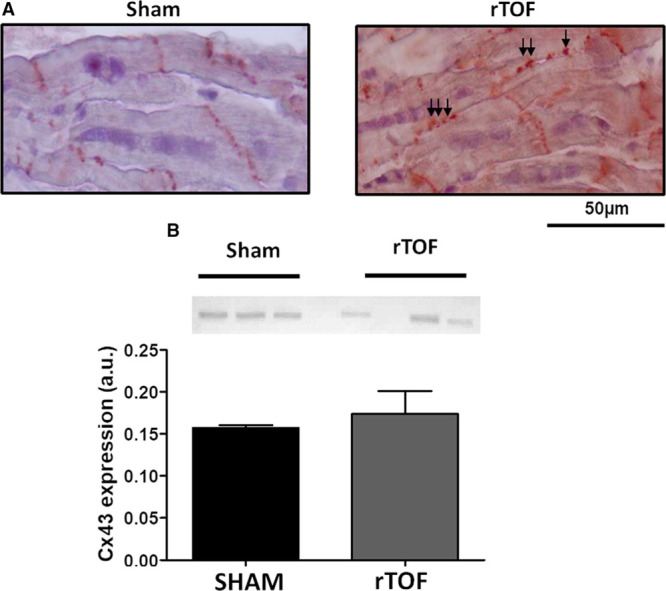
**Membrane localization and expression of Connexin-43 in Sham and repaired tetralogy of Fallot (rTOF) left ventricles (LVs).** **A**, Immunohistochemistry for Cx43 (connexin-43) performed on 8 µm LV base tissue sections revealed Cx43 Lateralization (arrows) in rTOF LVs as opposed to Sham LVs in which Cx43 was localized at the intercalated discs. **B**, Cx43 expression was unchanged in rTOF LVs compared with Sham. Data are means±SD. Sham, n=3; rTOF, n=3.

**Figure 7.**
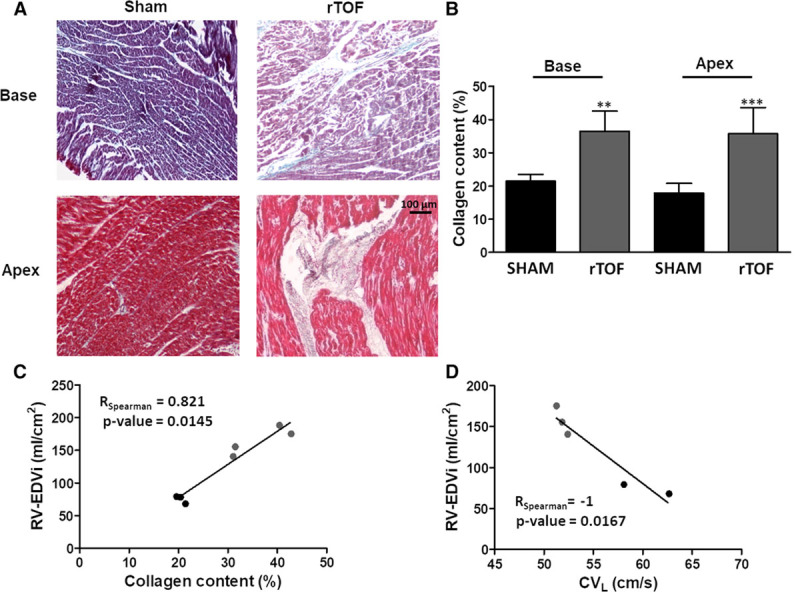
**Collagen content in Sham and repaired tetralogy of Fallot (rTOF) left ventricles (LVs).** **A**, Representative histological sections of Sham and rTOF LVs stained with Masson trichrome. In addition to diffuse fibrosis, large fibrotic regions are visible in the LV base and apex of rTOF pigs. **B**, Collagen content, expressed as a percentage of total section area, was increased in rTOF LV base and apex compared with Sham. **C**, A positive correlation was found between LV collagen content and right ventricular end-diastolic volume indexed to body surface area (RV-EDVi), and (**D**) a negative correlation was found between RV-EDVi and LV longitudinal conduction velocity (CV_L_). Data are means±SD. Sham, n=4; rTOF, n=4. **P<0.01, ***P<0.001.

LV epicardial activation maps are shown in Figure 4A. The blue represents early activation time and red late activation time (5 ms spaced isochrones). Longitudinal conduction velocities were decreased in rTOF compared with Sham pigs (51.27±0.57 versus 65.37±6.76 cm/s; Figure 4B), but transverse conduction velocities were unchanged (30.19±1.97 versus 27.34±3.08 cm/s; Figure 4C). This resulted in a trend (*P*=0.057) for an alteration of the anisotropy of propagation as indicated by the decreased longitudinal-to-transverse conduction velocity ratio (Figure 4D). Longitudinal conduction velocity was significantly decreased in rTOF at pacing frequencies ≤1.5 Hz, resulting in shallower longitudinal conduction velocity restitution curves (Figure 5A), whereas transverse conduction velocity restitution properties were unchanged (Figure 5B). Lateralization of connexin-43 was found in rTOF pigs (Figure 6A), but protein level of connexin-43 was not significantly altered in rTOF pigs compared with Sham (Figure 6B). Masson trichrome staining showed large fibrotic regions at the base and apex of the LV in rTOF pigs (Figure 7A). The quantification of collagen content confirmed the presence of a significant increase in diffuse fibrosis in rTOF LV sections compared with Sham (Figure 7B). Interestingly, LV collagen content correlated with RV end-diastolic volume indexed to BSA indicating a potential role for adverse ventricular-ventricular interactions in rTOF LV fibrosis (Figure 7C). Moreover, a negative linear correlation was found between RV end-diastolic volume indexed to BSA and LV longitudinal conduction velocity (Figure 7D).

### Arrhythmias

During our dynamic restitution protocols in pefused LV wedges, we found 4.2 arrhythmic events per pig on average in the rTOF group (all of which were sustained ventricular tachycardia of focal origin) compared with 1.8 in the Sham group (all of which were sustained ventricular tachycardia of focal origin apart from 1 ventricular fibrillation episode; *P*=0.107). Analysis of these arrhythmic events revealed that arrhythmias occurred at lower stimulation frequencies in rTOF than Sham LVs (Sham, 5±0 vs rTOF, 3.6±0.8 Hz; *P*=0.047). Interestingly, we found that the 2 rTOF LVs with the highest number of arrhythmic events (n=6 events for each) had the lowest stimulation frequency thresholds for arrhythmia induction (3–3.75 Hz), and arrhythmias were preceded by APD alternans (at 2–2.5 Hz) in these preparations (Figure III in the Data Supplement).

## Discussion

In the present study, we have investigated the hypothesis that ventricular-ventricular interactions may lead to deleterious LV remodeling in an rTOF porcine model mimicking residual pulmonary stenosis, regurgitation, and infundibular scar. We found, despite no apparent change in LV function, significant alterations of LV electrical and structural properties, reminiscent of the proarrhythmic substrate typically found in heart failure. These findings may have important implications for risk stratification and prevention of ventricular arrhythmias in patients with rTOF.

### Ventricular Interactions and LV Remodeling in rTOF

The mechanisms underlying LV remodeling in rTOF are relatively unexplored, but emerging evidence highlights the role of the adverse ventricular-ventricular interactions^20^ and RV dysfunction in driving this process.^21^ In line with these observations, we found in our animal model an increase in myocardial collagen content and a decrease in conduction velocity that correlated with RV end-diastolic volume, although this will need to be further explored in future studies. Studies have shown that RV dilatation, especially at the apex, may be important in leading to altered LV geometry and consequently to abnormal apical rotation and decreased LV efficiency.^22^ The impact of RV overload on LV function is variable depending on the type of overload (volume versus pressure) and the stage (acute versus chronic) and the role of the interventricular septum has been highlighted.^23^ Although chronic volume overload is expected to result in depressed LV ejection fraction, this is not the case in RV pressure overload until advanced pathological stages. These interactions may become more complicated in patients with rTOF because both RV volume overload and pressure overload may take place. In rTOF pigs, we observed both an end-diastolic and -systolic leftward septal shift (Figure IV in the Data Supplement) with no significant change in LV ejection fraction. The leftward septal shift may lead to altered strain and stress patterns, especially at the septal insertion points with the LV free wall. Thus mechanoelectrical coupling is likely to contribute to LV electrophysiological remodeling in rTOF. In patients with rTOF, Ghai et al^24^ have shown that LV systolic dysfunction plays a role in sudden cardiac death. It is interesting to note that in our animal model, the proarrhythmic LV remodeling occurs before functional changes.

### Electrical Remodeling of the rTOF LV

So far, experimental studies related to rTOF electrical remodeling and arrhythmias primarily focused on the RV.^11,12,25,26^ However, recent works on rodent models of RV pressure overload have provided evidence for ventricular-ventricular interactions and arrhythmogenic remodeling of the LV.^18^ Here, we find a significant APD_80_ prolongation in the LV epicardium and endocardium of rTOF hearts. Furthermore, we observed increased APD dispersion on the LV epicardium of rTOF animals, although no significant changes were found in transmural APD dispersion as has been previously described in various animal models of LV hypertrophy and heart failure. Finally, longitudinal conduction velocity was reduced in the rTOF group and was associated with connexin-43 lateralization and increased interstitial fibrosis.

Previous studies in the monocrotaline rat model of pulmonary hypertension showed a modest prolongation of the APD^17^ and effective refractory period^18^ throughout the LV when compared with the RV. This was mainly attributed to changes in the expression levels of the main potassium channels responsible for repolarization in the rat myocardium. In our study, the prolongation of APD_80_ was pronounced, albeit not homogenous, in the entire LV. Indeed, APD_80_ was prolonged at the apex and mid free wall but not in the base of LV (Figure II in the Data Supplement). This resulted in elevated epicardial APD_80_ heterogeneity, which may be because of heterogeneous stress and strain patterns induced by the RV dilatation and dysfunction.

### Structural Remodeling of the rTOF LV

Although we did not find evidence for LV hypertrophy, we did observe microstructural remodeling. Histological sampling was performed at the base and apex of the LV. The large amount of LV fibrosis found in these regions may be related to altered strain and stress patterns leading to local LV stretch especially as LV collagen content correlated with RV end-diastolic volume indexed to BSA. Myocardial stretch is known to promote fibrosis.^27^ Little attention has been paid to the structural remodeling of the LV in patients with rTOF. Nevertheless, in recent clinical studies, including various cohorts of rTOF patients (ranging from children to adults having undergone different surgical procedures), an increase in extracellular volume, indicative of diffuse myocardial fibrosis, was found not only in the RV but also in the LV free wall.^28^ Furthermore, LV structural changes were found to correlate with RV remodeling and the occurrence of arrhythmias.^15^ Our animal study not only reproduces these recent clinical findings but also shows that fibrosis is associated with significant electrophysiological alterations, including conduction slowing and increased dispersion of repolarization, which could play a major role in the onset and maintenance of ventricular arrhythmias.

### Arrhythmias in the rTOF LV

Electrical and structural alterations found in rTOF LVs, including longer, heterogeneous repolarization, together with slower conduction and fibrosis generate a substrate for arrhythmias. While assessing electrical restitution properties by progressive increases in stimulation frequency, we did observe arrhythmias in our preparations. Our results suggest an increased vulnerability to arrhythmias at increased heart rates in rTOF pigs. Interestingly, we found that in the most arrhythmogenic rTOF LVs, arrhythmias were preceded by APD alternans. T-wave alternans is a well-described risk marker for sudden cardiac death^29^ and has recently been described in the RV of an rTOF dog model.^26^

### Translational Perspective

The LV remodeling found in our preclinical model of rTOF involved a prolongation and dispersion of repolarization, a slower conduction velocity, and increased collagen deposition which correlated with RV dilatation. These results suggest a role for the LV in arrhythmogenesis in rTOF as a consequence of adverse ventricular-ventricular interaction at the tissue level.

Even though LV hypertrophy and dysfunction were not observed in this porcine model, our study shows that an arrhythmogenic substrate may develop in the LV early on after surgical repair. Further clinical studies in patients with rTOF are needed to assess the role of RV-LV interactions and the time course of LV electrophysiological remodeling. These may lead to the development of antiarrhythmic therapies aiming at the LV or suggest a timing for pulmonary valve replacement to reduce RV dilatation and deleterious ventricular-ventricular interactions.

### Limitations

The present animal model reproduced postoperative sequelae in TOF, such as hemodynamic and electrophysiological properties, in healthy pigs without underlying congenital disease. In this context, factors independent of surgery, such as genetic substrate^30^ and hypoxemia,^31^ may influence the proarrhythmic ventricular remodeling. Moreover, we did not reproduce the ventricular septal defect correction in our animals because this intervention considerably increased mortality. Although ventricular septal defect patch is known to support reentrant circuits in patients with rTOF, this is unlikely to affect LV electrophysiological remodeling, which was the main mechanism investigated in this study. The protocol duration may seem short relative to the decades it takes to reach RV dysfunction and arrhythmias in patients with rTOF. However, in a former study using this animal model,^12^ we found RV dysfunction was similar to that observed in patients with rTOF. Moreover, the aim of the present study was to identify early remodeling of the LV, which could be therapeutically targeted or used as marker to predict the optimal timing of pulmonary valve replacement. It must be noted that a longer protocol may further enhance LV remodeling, lead to LV dysfunction, and increase arrhythmogenicity in our pig model. However, it is also possible that our study was not powered enough to detect a significant difference in arrhythmia inducibility.

### Conclusions

We found a significant remodeling of LV repolarization and conduction properties in the absence of LV dysfunction in a porcine model reproducing TOF repair consequences. Fibrosis was associated with these significant electrophysiological alterations and could play a major role in the onset and maintenance of ventricular arrhythmias in corrected TOF patients.

## Acknowledgments

Drs Benoist, Roubertie, Quesson, Cochet, Haïssaguerre, Rooryck, Bordachar, Thambo, and Bernus conceived and designed the experiments; Dr Benoist, Dr Dubes, Dr Roubertie, Dr Gilbert, S. Charron, M. Constantin, Dr Elbes, D. Vieillot, and Dr Quesson performed the experiments; Dr Benoist, Dr Dubes, Dr Gilbert, S. Charron, and Dr Bernus analyzed the data; Drs Dubes, Benoist, and Bernus wrote the paper.

## Sources of Funding

This work was funded by the Agence Nationale de la Recherche (ANR-10-IAHU04-LIRYC), the European Research Council under the Seventh Framework Programme of the European Union (FP/2007-2013) (grant agreement No. ERC-2012-AdG_20120314), and the FP7 research and innovation programme of the European Union under the Marie Sklodowska-Curie (FP7-PEOPLE-2012-IRSES), grant agreement No. 317766. Dr Benoist was funded by a Fondation Recherche Médicale fellowship, and Dr Gilbert was funded by a Marie Skoldowska-Curie fellowship from the European Research Council (grant agreement No. 715093). There are no relationships with the industry.

## Disclosures

None.

## Supplementary Material
