# Supplementary material for: Arrhythmogenic Remodeling of the Left Ventricle in a Porcine Model of Repaired Tetralogy of Fallot
Source: Circ Arrhythm Electrophysiol. 2018 Oct 10;11(10):e006059. doi: 10.1161/CIRCEP.117.006059 (PMC6553519; doi:10.1161/CIRCEP.117.006059)
Supplement: Supplementary file 1 [file hae-11-e006059-s001.pdf]

SUPPLEMENTAL MATERIAL

Methods

Animal Model

Large White newborn piglets (<12 kg) were used in accordance with the "Guide for the Care and Use of Laboratory Animals" (NIH Publications No. 85-23, revised 1996) and with approval of the local ethical committee.

The animals were sedated with ketamine (10 mg/kg, intramusculary (IM), Vibrac) and acepromazine (0.1 mg/kg, IM, Vetoquinol). Anesthesia was induced with sodium pentobarbital (5 mg/kg, intravenously (IV) Ceva) and maintained with isoflurane (2% in 100% oxygen, Vibrac) following endotracheal intubation.

A left thoracotomy between the fourth or fifth intercostal space was performed to access the heart. The pulmonary artery was clamped longitudinally, a 2 cm incision was made across the pulmonary annulus, and 2 pulmonary valve leaflets were excised. A polytetrafluorethylene patch was sewn across the pulmonary infundibulum and a loosely tied tape was passed around the artery (2 cm diameter).

Six animals underwent repaired surgery (rTOF) and five control animals were sham-operated (Sham) for which only the lateral thoracotomy was performed.

Animals were then studied at 23 ± 1 weeks post-surgery to assess cardiac function and electrophysiological remodeling.

Cardiac Magnetic Resonance

Upon return in the lab, animals were sedated and anaesthetized as explained above. A cardiac magnetic resonance (CMR) was performed using a Siemens MagnetomAvanto 1.5T MRI scanner (Erlangen, Germany). Pigs were placed in dorsal recumbancy and ECG leads placed after shaving and hair removal (Veet, Reckitt Benckiser, UK) and the telemetric vector electrocardiogram was used for gating of the Siemens CMR sequences. All images were taken during end-expiratory ventilation stop. Localizer views

were followed by right-ventricular short-axis TrueFISP cine-image stacks (from the cardiac apex to the level of the pulmonary valve) under manual-breathhold. For pulmonary artery flow measurement phase-contrast MRI was carried out under manual-breathhold with the velocity encoding (VENC) set to 130 cm/s and the acquisition plane set parallel to and 1 cm above the pulmonary valve plane. CMR analysis was performed by a single investigator. Quantification of ventricular function was carried out using the Siemens syngo ARGUS Ventricular Function software package with manual drawing of a right ventricular endocardial contour and automatic LV segmentation from which end-systolic, end-diastolic, stroke volume and ejection fraction were determined. These values where indexed to body surface area (BSA) using the equation from Kelley et al [1]. The pulmonary regurgitation fraction was calculated using the Siemens syngo ARGUS Flow software package with the rephased images used to draw the analysis ROI and quantification calculated from the phase images. Pulmonary regurgitation fraction was calculated as the ratio of pulmonary retrograde to antegrade flow volume (expressed as a percentage).

After the MRI acquisition, pigs were euthanized by injection of sodium pentobarbital (10 mL from 200 mg/mL stock) and their hearts were rapidly excised. The aorta was cannulated and rinsed with an ice-cold cardioplegic solution containing (in mM): 110 NaCl, 1.2 $CaCl_2$, 16 KCl, 16 $MgCl_2$, 10 $NaHCO_3$, 9 Glucose supplemented with Heparin (2500 UI/L). The hearts and the LV were dissected and weighed.

Optical mapping of anterior LV wedges

The left anterior descending (LAD) coronary artery was cannulated and perfused with cardioplegic solution while suturing arterial leaks. Following suturing, the wedge was installed in a bath (37°C) and perfused at constant flow rate (20 mL/min) with modified Krebs-Henseleit solution containing (in mM): 130 NaCl, 4 KCl, 24 $NaHCO_3$, 1.2 NaH_2PO_4, 1 $MgCl_2$, 5.6 Glucose, and 1.8 $CaCl_2$. The solution was gassed with 95% O_2 and 5% CO_2 (pH 7.4) and the perfusate was maintained at 37°C. Motion artifacts induced by contraction were suppressed using the electro-mechanical uncoupler blebbistatin (10 µM, Enzo Life Sciences). Following staining with the voltage-sensitive dye di-4ANEPPS (10 µM in bolus, Biotium), the LV wedge was excited by LED illumination at 530 nm (Cairn Research). Fluorescence

emitted (optical signal) from both the epicardium and endocardium was filtered at 650 ± 50 nm and recorded simultaneously at 1 kHz with two MicamUltima CMOS camera (SciMedia USA Ltd) at 1 mm spatial resolution. Optical signals were filtered (3 x 3 spatial and 3 x 3 cubic filter) and background fluorescence was subtracted.

The wedge was paced from 1 Hz to 5 Hz by 0.5 Hz increments with a 5 ms pulse on the base of epicardium to investigate restitution properties. Ventricular arrhythmias occurring during the dynamics restitution protocol were monitored and were classified according to their type (ventricular tachycardia or fibrillation), duration and stimulation frequency threshold required for induction. Sustained arrhythmias were defined as lasting for at least 30s and requiring external defibrillation for termination (30J). Action potential duration was measured at 80% of repolarization (APD_{80}) in 5 x 5 mm regions at the base, the mid-free wall, the apex of the anterior LV. The maximum slope of the APD_{80} restitution curves was determined after monoexponential curve fitting. APD_{80} dispersion was calculated as the difference between the 95th and the 5th percentile of the APD_{80} distribution across the LV. Repolarization time dispersion was calculated by subtracting minimal from maximal repolarization time over the whole field of view. The effective refractory period (ERP) was measured by an S1-S2 protocol, consisting of continuous 10 S1 stimuli at 1000 ms cycle length followed by an additional S2 stimulus at gradually decreasing coupling intervals. The last coupling interval that produced an action potential was defined as the ERP. Longitudinal conduction velocity (CV_L) and transverse conduction velocity (CV_T), were measured along the directions of fastest and slowest propagation velocity from the stimulus point.

Histology

Tissue samples (1.5 x 1.5 cm) were collected from the apex and the base of the LV and fixed in 4% paraformaldehyde (Sham, rTOF N=4). Samples were immersed in ethanol at increasing concentrations (70%, 90%, 100%) for dehydration, rinced with toluene, embedded in paraffin and stored at -20°C until use. Transmural sections (8 μm) were stained with Masson’s Trichrome (HMS70, Thermo Scientific). Slides were examined at 10X magnification on a Nikon Eclipse 80i equipped with a CDD camera.

Analysis was performed blindly on 3 sections per animal by color thresholding using ImageJ software. Blue-stained areas corresponding to collagen were expressed as a percentage of the section total area. Perivascular and epicardial collagen staining were excluded from the quantification.

Immunohistochemistry

After deparaffinization and dehydration, the transmural sections were treated with 3% H_2O_2 at -20°C for 10 minutes to quench endogenous peroxidase activity. Then all sections were treated at 100°C with sodium citrate buffer, pH 6 (Dako) for 20 minutes for antigen retrieval. After blocking endogene avidine and biotine (Dako), sections were incubated with 10% donkey serum for 10 minutes to block nonspecific reactions. To finish, sections were incubated with anti-connexin 43 antibodies (mouse polyclonal, 1:250, Millipore) overnight at 4°C. After rincing with Phosphate Buffer Saline, sections were incubated with biotinylated secondary antibodies and the conjugated enzyme streptavidine peroxydase. Immunoreactive cells were observed with the chromogene3 Amino 9 ethyl-carbazole and then counterstained with Hematoxylin. Slides were examined at a 40X magnification on a Nikon Eclipse 80i equipped with a CDD camera.

Western Blots

Epicardial and endocardial tissue layers were dissected from LV free wall and snap frozen in liquid nitrogen. All samples were stored at -80°C until protein extraction. Tissue sample were homogenized and sonicated in RIPA buffer (Sigma Aldrich) completed with proteases and phosphatases inhibitor cocktails (Sigma Aldrich). Proteins were extracted by centrifugation at 12 000 g for 15 min at 4°C. Protein concentration was determined by bicinchonic acid assay (Thermo Scientific) and before separation (50 µg) on 12% acrylamide gels (TGX Stain-Free precast gels, Bio-Rad) under reducing conditions. Following semi-dry transfer (Trans-blot Turbo, Bio-Rad), PVDF membranes were incubated overnight at 4°C with the appropriate primary antibodies (Connexin-43, 1:500, Sigma-Aldrich) followed by incubation with secondary antibodies (Goat anti-rabbit, 1:2000, Bio-Rad). Total hybridized proteins were imaged

under UV light and Connexin-43 specific signals were revealed by enhanced chemiluminescence (Thermo Scientific). Western blot quantification was performed using ImageJ (NIH). Specific immunosignals were normalized to the total protein content of each lane.

Data analysis

Data are presented as mean ± SD. Differences were determined using Mann-Whitney tests, one-way ANOVA with a Holm-Sidak multiple comparison test or its non-parametric equivalent, and Spearman correlation analysis as appropriate using SigmaStat software. A p-value of $P < 0.05$ was considered significant.

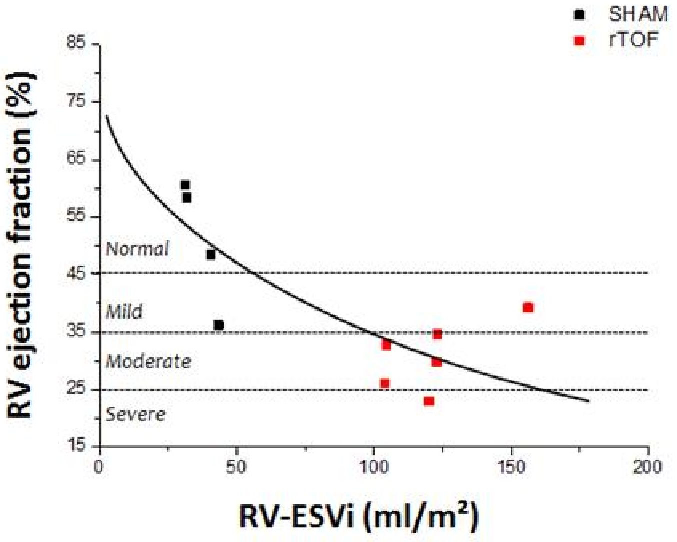

Supplemental Figure 1 – Right ventricular dysfunction severity grading in rTOF pigs. Relationship between right ventricular end-systolic volume normalised to BSA (RV-ESVi) and RV ejection fraction obtained by MRI. Sham N=4, rTOF N=6.

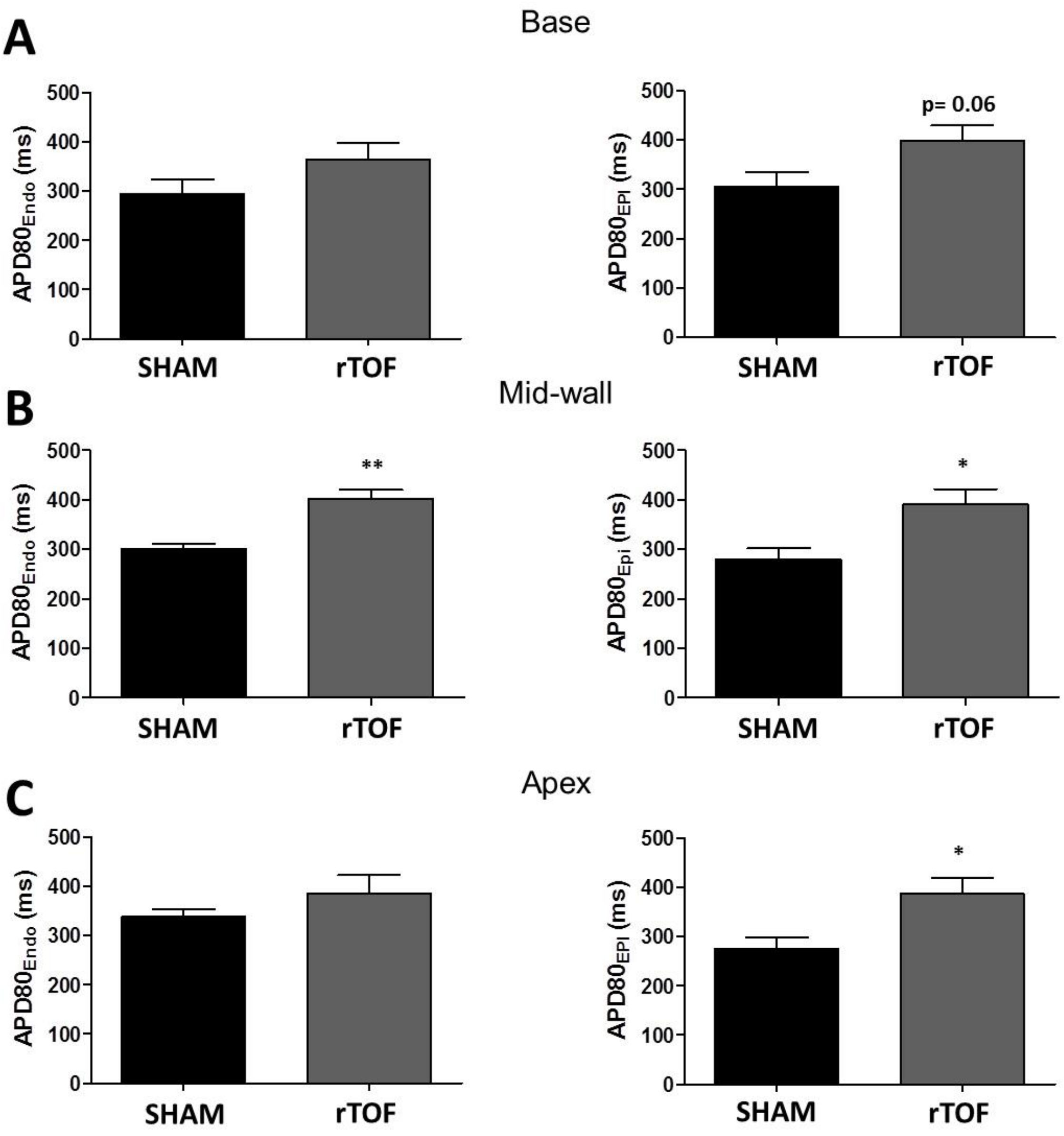

Supplemental Figure 2 – Regional action potential duration in Sham and rTOF anterior LVs. Epicardial and endocardial action potential duration (APD_{80}) was measured in the base **(A)**, mid-wall **(B)**, and apex **(C)** of LVs stimulated at 1 Hz. APD_{80} was increased in the mid-wall epicardium and endocardium as well as in the epicardium apex. $^{*}P < 0.05$, $^{**}P < 0.01$ Sham n=5, rTOF n=6.

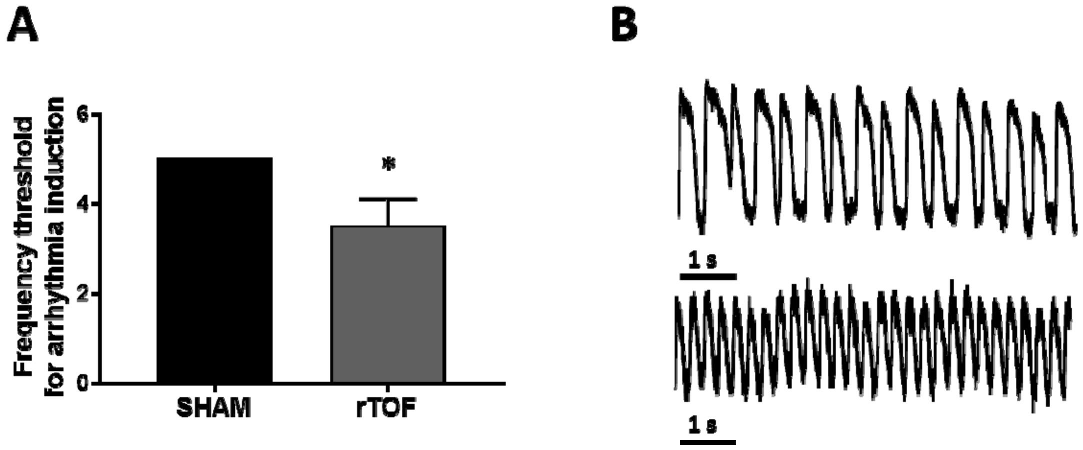

Supplemental Figure 3 – Stimulation frequency threshold for arrhythmia induction and action potential duration altenans. The frequency threshold for sustained ventricular tachycardia (VT) or fibrillation (VF) during dynamic restitution protocols was lower in rTOF than Sham in perfused LV preparations **(A)**. Traces of action potential duration alternans (upper panel) and ventricular tachycardia (lower panel) in a rTOF LV **(B)**. Alternans were observed at low stimulation frequency (2 - 2.5 Hz) in 2 rTOF LV preparations with the highest number of arrhythmic events. Further increase in stimulation frequency led to VT in these preparations. *P < 0.05 Sham n=5, rTOF n=6

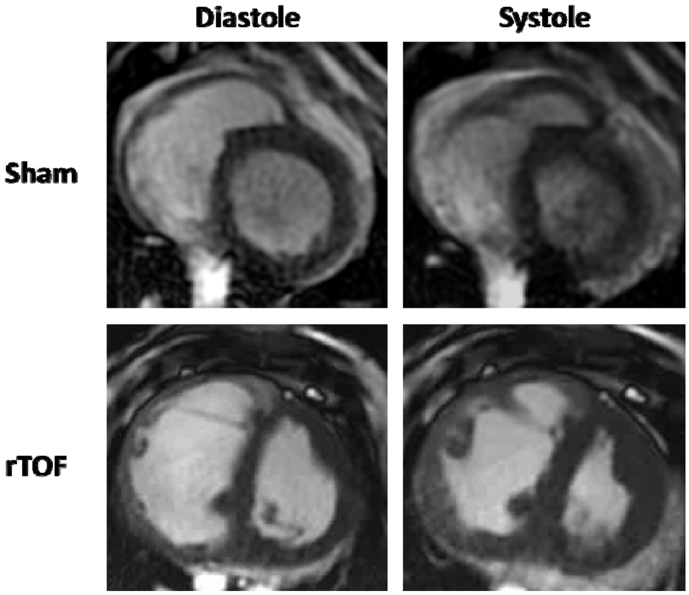

Supplemental Figure 4 – Short axix images of a Sham and rTOF heart during a cardiac cycle.

Cardiac MRI equatorial short axis slices obtained from a cine loop of the heart of a Sham and a rTOF pig showing diastole (left panel) and systole (right panel). Note the presence of a major leftward septal shift in a rTOF pig.

REFERENCES

1. Kelley KW, Curtis SE, Marzan GT, Karara HM, Anderson CR. Body surface area of female swine. *J Anim Sci*. 1973;36:927-930
