# Supplementary figures and images for: Arrhythmogenic Remodeling of the Left Ventricle in a Porcine Model of Repaired Tetralogy of Fallot
Source: Circ Arrhythm Electrophysiol. 2018 Oct 10;11(10):e006059. doi: 10.1161/CIRCEP.117.006059 (PMC6553519; doi:10.1161/CIRCEP.117.006059)

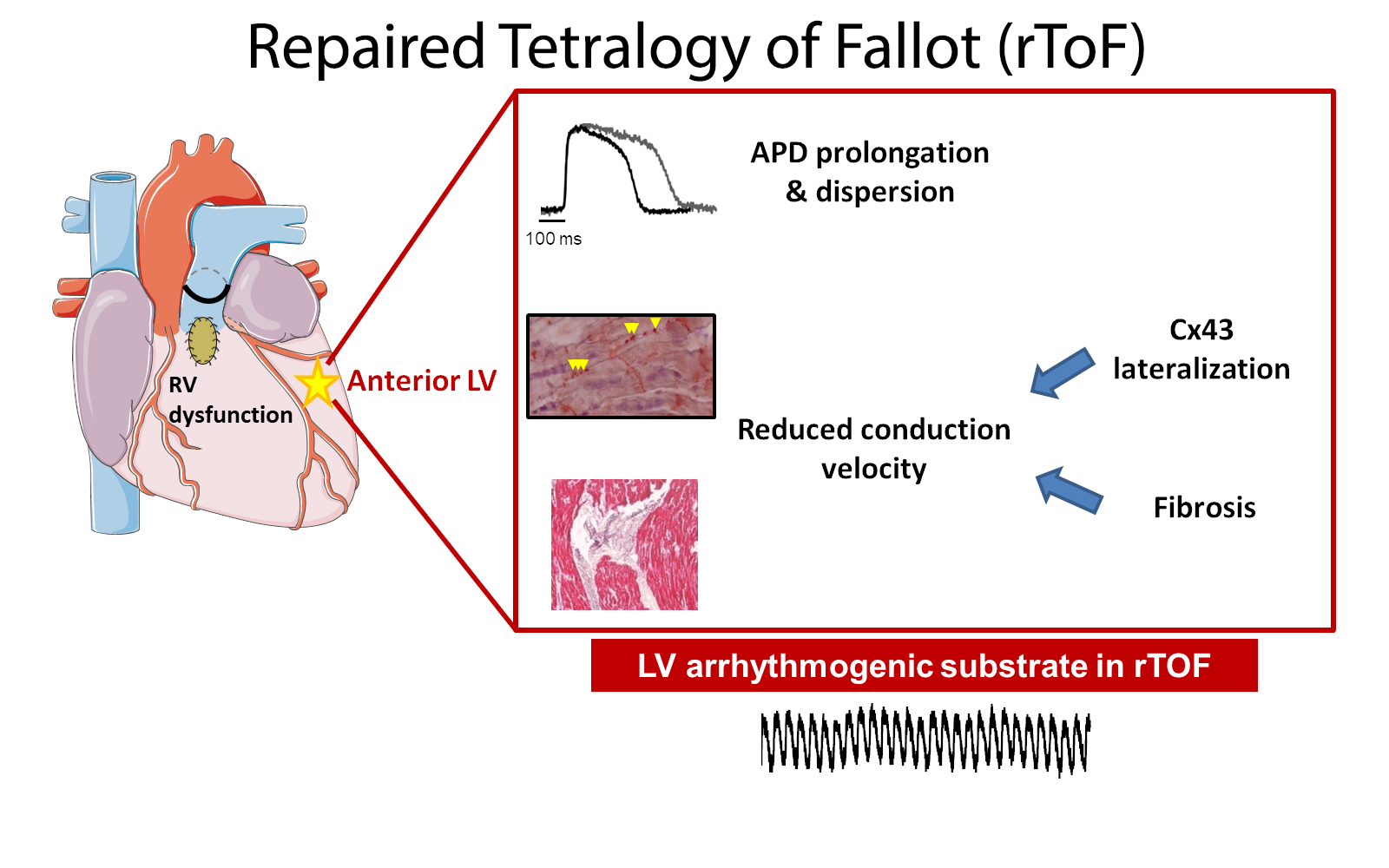

Supplement: Supplementary file 2 [file hae-11-e006059-s002.tif]
